# Novel enterprises digital transformation influence empirical study

**DOI:** 10.1371/journal.pone.0296693

**Published:** 2024-01-12

**Authors:** Xiaowen Sun, Wenjing Sun, Zheng Wang

**Affiliations:** 1 Department of Economics, Beijing University of Posts and Telecommunications, Beijing, China; 2 School of Medical Economics and Management, Anhui University of Chinese Medicine, Hefei, China; 3 Key Laboratory of Data Science & Innovative Development of Traditional Chinese Medicine, Philosophy and Social Sciences of Anhui Province, Hefei, China; Universiti Malaysia Sabah, MALAYSIA

## Abstract

With the rapid development of technologies such as cloud computing and big data, various levels of government departments in the country have successively introduced digital subsidy policies to promote enterprises’ digital transformation. However, the effectiveness of these policies and their ability to truly achieve policy objectives have become pressing concerns across society. Against this backdrop, this paper employs a moderated mediation effects model to empirically analyze the incentive effects of financial subsidies on the digital transformation of A-share listed manufacturing companies in the Shanghai and Shenzhen stock markets from 2013 to 2022. The research findings indicate a significant promotion effect of financial subsidies on the digital transformation of manufacturing enterprises, especially demonstrating a notable incentive impact on the digital transformation of large enterprises, non-asset-intensive enterprises, technology-intensive enterprises, and non-labor-intensive enterprises. However, the incentive effect on the digital transformation of small and medium-sized enterprises (SMEs), asset-intensive enterprises, non-technology-intensive enterprises, and labor-intensive enterprises is not significant. Notably, the expansion of financial subsidies positively influences the augmentation of R&D investment within manufacturing enterprises, subsequently providing indirect encouragement for their digital transformation. Additionally, the incorporation of the degree of marketization implies its potential to moderate both the direct and indirect impacts of financial subsidies on enterprise digital transformation. This study enriches the research on the mechanism of the role of financial subsidies in digital transformation and provides empirical evidence on how market participation influences the effects of financial subsidies, thereby assisting policymakers in comprehensively understanding the impact of financial subsidy policies on different types of enterprises.

## 1. Introduction

As the swift advancement of information technology converges with the momentum towards global economic integration, the journey towards digital transformation has become essential for companies seeking to enhance efficiency, adaptability, and innovative capabilities. This transformation transcends mere technological updates, involving significant shifts in the entire operational frameworks, structural organization, and competitive stance in the market. Despite the significant consensus on the importance of digital transformation, the academic community has yet to establish a precise definition. A number of academics argue that the essence of digital transformation lies in the cultivation of digital technologies and the necessary supportive skills. This approach is key in forming a dynamic digital business model. [1].

The manufacturing industry, as the backbone of the real economy and a core engine for long-term stable economic growth, is particularly vulnerable and in need of transformation. Although China’s manufacturing industry boasts a large scale and a complete system, issues such as being large but not strong and comprehensive but not optimal are prominent. Against the backdrop of the gradual weakening of China’s manufacturing cost advantages, the industry faces challenges such as weak independent innovation capabilities and inefficient production management. Digital transformation is considered a crucial means to enhance the competitiveness of the manufacturing industry, presenting numerous opportunities, including improving production efficiency, achieving product innovation, and strengthening supply chain collaboration [2–5].

However, in the current digital economy landscape, the manufacturing industry is confronted with urgent challenges in digital transformation. High costs and long payback cycles of technological innovation reduce the private benefits brought about by enterprise innovation, diminishing enthusiasm for research and development and causing market failures. To address this issue, the government has introduced a series of policies, including providing financial subsidies, tax incentives, and improving intellectual property protection systems to stimulate enterprise innovation activities. Yet, the specific role of government financial policies in digital transformation remains unclear. Additionally, government support carries inherent risks, such as potential overcapacity and dependence on government incentives. Therefore, an in-depth study of the actual role of government financial policies in digital transformation, as well as the pain points and future optimization directions for enterprise digital transformation, will contribute to enhancing policy implementation efficiency and guiding the manufacturing industry towards a digital future.

Currently, research in academia regarding the relationship between financial subsidies and enterprise digital transformation predominantly focuses on the impact and effectiveness of financial subsidies on digital transformation. However, the conclusions drawn from these studies are highly debated and have not reached a consensus. On one hand, financial subsidies, driven by policy and time sensitivity, can provide a stable cash flow, supporting enterprises’ digital transformation efforts within a specified timeframe. Consequently, this approach eases financial limitations, diminishes the costs and risks associated with transformation, and motivates companies to amplify their endeavors. This includes increasing investments in digital infrastructure, hiring skilled digital professionals, and embracing advanced digital technologies. On the other hand, financial subsidies, with specific purposes, may also entail higher institutional costs. Consequently, enterprises may divert subsidy funds towards "strategic transformation" rather than substantive transformation, leading to inefficient utilization of financial resources, hindering the progress of digital transformation [6–8].

Furthermore, in the exploration of the mechanisms through which financial subsidies affect enterprise digital transformation, some researchers have found that alleviating financing constraints, increasing R&D investments, and promoting innovation output are crucial channels through which financial subsidy policies influence enterprise digital transformation. Thus, financial subsidies may to some extent stimulate the digital transformation of manufacturing enterprises. Moreover, due to the differences among manufacturing enterprises in terms of scale, industry, ownership, and region, the policy effects of financial subsidies may exhibit heterogeneity [9–13].

In summary, the current research on financial subsidies and digital transformation exhibits the following shortcomings: First, existing studies on enterprise digital transformation primarily concentrate on macro and intermediate levels, with limited research at the micro level [14–16]. Second, there is still significant debate about the impact of financial subsidy policies on enterprise digital transformation, making it difficult to reach a consensus. Additionally, there is a scarcity of scholarly research on the mechanisms through which financial subsidies affect enterprise digital transformation, leaving questions about how government subsidies influence digital transformation pathways largely unanswered. Third, there is limited research on the heterogeneity of enterprises concerning government subsidies and digital transformation, particularly in terms of differences in enterprise size and nature.

This research explores the correlation between monetary grants and the digital transformation in the manufacturing industry. It analyzes data sourced from manufacturing firms listed on the Shanghai and Shenzhen stock exchanges over the period from 2013 to 2022. The empirical analysis employs panel data to investigate the stimulating effect of financial subsidies on internal digital transformation within enterprises. The study systematically expounds the influence of marketization level, mechanisms, and regulation. This research contributes in several ways: First, this research enriches the existing measures of digital transformation. It combines manual curation and computer text mining methods to select digital transformation indicators for assessing the level of digital transformation in manufacturing enterprises. Second, it enhances the research on the mechanisms of financial subsidies’ impact on digital transformation. When analyzing the policy transmission process, we clarify both the direct and indirect effects of financial subsidies on digital transformation in manufacturing enterprises, with R&D investment acting as an intermediary variable, aiding in explaining the policy transmission mechanisms. Third, this research provides empirical evidence on how market participation affects the effectiveness of financial subsidies. We utilize an adjusted mediation effect model, innovatively incorporating the level of marketization as a moderating variable to investigate the influence of market participation on the policy transmission pathways of financial subsidies. Finally, this research assists policymakers in comprehensively understanding the impact of financial subsidy policies on different types of enterprises. Recognizing the varying sizes and natures of these enterprises, we examine the differential impacts of government financial subsidies on digital transformation. This analysis offers a comprehensive understanding of the ways in which these policies affect different types of businesses.

## 2. Theoretical analysis and research hypothesis

### 2.1 Direct effect of financial subsidies on enterprise digital transformation

Financial subsidies refer to specific subsidies provided by specialized funds within the government to enterprises or individuals with the aim of achieving specific political and economic objectives. The impact of financial subsidy factors on enterprise digital transformation has been widely acknowledged by the majority of scholars. However, there is still considerable divergence in the conclusions drawn, and a consensus has yet to be reached. Current relevant research can be mainly categorized into two major viewpoints.

One perspective is the "promotion theory" of financial subsidies. The theoretical basis supporting this viewpoint includes the following aspects: Firstly, financial subsidies can offset the positive externalities associated with the digital transformation process. Given that digital transformation generates strong positive externalities, where the private returns from this transformation are lower than the corresponding social returns, private investments tend to fall below the socially optimal level [17]. According to Kleer (2010), the government’s provision of financial subsidies can alleviate the positive externality cost pressure brought by digital transformation and further promote enterprises to achieve digital transformation and upgrading [18]. Research has found that financial assistance from fiscal science and technology expenditure can help improve the financial condition of enterprises. The government’s financial support to enterprises is a kind of behavior to screen high-efficiency and high-potential enterprises, which can send a positive signal to outside investors. In addition, it can guide financial resources to flow more targeted to specific enterprises, providing a solid financial basis for their research and development, innovation and transformation [19–21]. Secondly, financial subsidies can ameliorate the financial constraints faced by enterprises. Hamberg initially found, through studying the impact of U.S. tax policies on innovation levels, that government subsidies can address the funding shortage in enterprises’ research and development activities [22]. Wang Gang et al. propose that government innovation subsidies are targeted and can directly make up for the resource deficiencies of enterprises [23]. However, when government support is insufficient, it will limit the digital transformation of enterprises. Scholars like Wu and Chen et al., using empirical research based on data from Chinese listed companies, have reaffirmed the conclusions of Claudia and other scholars [24, 25]. It is beyond doubt that digital transformation requires substantial funding [26]. While enterprises prioritize using internally retained earnings, it is often insufficient, necessitating external financing to bridge the funding gap. Financial subsidies not only directly supplement the pathways of companies’ internal funds but also convey a reliable "government backstop" signal to the market. These signals attract investors to invest in enterprises, thereby facilitating the acquisition of cash flow required for purchasing the resources necessary for digital transformation. In summary, government financial subsidies, through both the direct effects of injecting funds and the indirect effects of signaling, provide guarantees for enterprise digital transformation in terms of internal and external funding channels, alleviating enterprises’ financing constraints.

Some argue for the "inhibition theory" of financial subsidies. According to this perspective, the lack of government oversight on how enterprises utilize subsidies, coupled with a limited understanding of the specific purposes of subsidies, may lead to funds being diverted for alternative uses. This diversion, in turn, diminishes the intended positive impact of financial subsidies on innovation. Moreover, there is a risk that enterprises might misconstrue subsidies, erroneously believing they can easily attain additional profits without engaging in research and development efforts [27, 28]. Li pointed out that financial subsidies encourage enterprises’ speculation in a disguised way [29]. As a consequence, this situation adversely impacts the enhancement of corporate innovation capabilities. To delve deeper into how financial subsidies influence corporate digital transformation, this research proposes the subsequent hypotheses:

**H1:** Financial subsidies play a direct motivating role in driving the digital transformation of manufacturing enterprises.

### 2.2 Indirect effect of financial subsidies on enterprise digital transformation

Financial subsidies represent a prominent mechanism to incentivize enterprises to enhance their innovation capabilities, and their effectiveness has become a focal point for scholars both domestically and internationally. Numerous studies from around the world have indicated that financial subsidies stimulate manufacturing enterprises to increase their investments in research and development (R&D). Hussinge conducted an empirical analysis, showing that German financial subsidies led to a significant over 30% increase in R&D expenditure for supported enterprises [30]. Consequently, it promoted the transformation and upgrading of German manufacturing enterprises. Similarly, research conducted by Czarnitzki et al. revealed that government-provided financial subsidies have a positive and profound impact on enterprise development, primarily evident in the stimulation of increased R&D investments, promotion of new product development, and the enhancement of profitability [31]. Studies by Lin and Ma consistently assert that government financial subsidies are effective in promoting R&D within an appropriate range [32, 33].

Furthermore, financial subsidies can indirectly influence the digital transformation of manufacturing enterprises by incentivizing increased R&D investments. The primary reasons behind this influence are as follows: First, financial subsidies are injected into enterprises in the form of cash flow [34], which aids in offsetting the costs and risks associated with R&D activities in the manufacturing sector. Government oversight of these specific subsidy funds fosters an incentive-compatible R&D environment [35], encouraging enterprises to intensify their R&D investments [36]. Subsequently, with the growth of R&D investments and the flourishing of R&D activities, manufacturing enterprises become more sensitive to cutting-edge technologies. This heightened awareness enables them to explore and integrate new technologies, thereby enhancing their technological capabilities and innovation potential [37]. As a result, this provides the essential hardware, technological support, and a solid foundation, facilitating their pursuit of digital transformation [38, 39].

Consequently, this study presents the following hypothesis:

**H2:** Financial subsidies indirectly promote the digital transformation of manufacturing enterprises by acting through R&D investment as an intermediary.

## 3. Model settings and data sources

### 3.1 model setting

This study seeks to explore the impact of financial subsidies on enterprise digital transformation. The benchmark regression model is formulated as follows:

$${Dig}_{i,t}=\alpha_{0}+\alpha_{1}{Sub}_{i,t}+\gamma_{1}{Controls}_{i,t}+\varphi_{i}+\theta_{t}+\varepsilon_{i,t}$$ (1)

Eq (1) explores the relationship between enterprises (*i*) across different years (*t*), considering factors such as enterprise digital transformation (*Dig*), financial subsidy status (Sub), and a set of relevant control variables (*Controls*). The model incorporates fixed effects for both enterprises (φ_*i*_) and years (θ_*t*_), along with a random disturbance term (ε_*i*,*t*_). A significant value of *α*_1_ would imply a substantial positive impact of financial subsidies on the digital transformation of manufacturing companies.

Expanding upon the baseline regression model in Eq (1), we develop a mediation regression model to investigate how financial subsidies influence the digital transformation of enterprises. This approach aims to assess the significance of the mediating effect without unnecessary repetition of details.


$${RD}_{i,t}=\beta_{0}+\beta_{1}{Sub}_{i,t}+\gamma_{2}{Controls}_{i,t}+\varphi_{i}+\theta_{t}+\varepsilon_{i,t}$$ (2)

$${Dig}_{i,t}=c_{0}+c_{1}{Sub}_{i,t}+c_{2}{RD}_{i,t}+\gamma_{3}{Controls}_{i,t}+\varphi_{i}+\theta_{t}+\varepsilon_{i,t}$$ (3)

In Eq (2) and Eq (3), RDit represents the intermediary variable, reflecting the level of R&D investment. The significance of β_1_ and *c*_2_, coupled with *c*_1_<α_1_, would suggest the efficacy of the mediating variable (degree of R&D investment), signifying the existence of a mediating effect.

### 3.2 Index selection

#### 3.2.1 Explained variable

The explained variable in this study is enterprise digital transformation (denoted as *Dig*). To accurately measure this variable, text mining methods were employed, utilizing annual reports of manufacturing companies from 2013 to 2022 as the data source. Building upon the research methodology of scholars such as Yuan [40], we further refined the process through manual identification of language expressions related to enterprise digital transformation. Subsequently, relevant keywords were selected using the Jieba function in Python, and keyword matrices were mapped and analyzed using Gephi software. Ultimately, we extracted the frequency of occurrence of each keyword in the annual reports of each company, thereby quantifying the extent of digital transformation.

#### 3.2.2 Core explanatory variable

The main explanatory variable in this study is financial subsidy (*Sub*). This study refers to the research conducted by Liu [41]. It derives the total amount of financial subsidies received by companies from the ’government grants’ disclosed in their annual reports. Furthermore, the government subsidy is subtracted from the tax preference to obtain the financial subsidy amount of the enterprise. Considering that the financial subsidy amount of the company in a certain year may be 0, and there are large differences in the financial subsidy amount of each company.We process the data with skewness to further reduce the impact of the outliers of the financial subsidy data on the analysis. In this study, the natural logarithm of the total financial subsidy plus one is taken, that is, Sub = ln(total amount of financial subsidies +1). In order to avoid the difference of order of magnitude between variables and make the regression coefficients sensitive for observation and comparison, the obtained results are scaled down by a factor of 100.

#### 3.2.3 Mediating variable

The mediating variable in this study is the degree of R&D investment (*RD*). There are various indicators for measuring the level of the R&D investment intensity. For instance, in a study by Dong on the impact of changes in tax rates on corporate research and development expenditure, the ln(1+ R&D investment) was used to gauge the level of the R&D investment intensity [42]. To eliminate the impact of differences in company size on the data, this study follows the approach of of Liu et al. [43]. The intensity of research and development investment is calculated as the ratio of total research and development investment to main operating revenue.

#### 3.2.4 Control variables

Reference to the practices of Tong, Liu, and Wang [44–46], we have incorporated various control variables based on existing domestic and international literature. These variables include:

**Asset-to-Liability Ratio (Lev):** The asset-to-liability ratio involves the relative proportion of debt to assets in a company’s capital structure. The capital structure of a company may influence its strategies and pace of digital transformation. Simultaneously, the level of the asset-to-liability ratio reflects a company’s capacity to bear risk; companies with high asset-to-liability ratios may be more susceptible to market changes and financial fluctuations, affecting their ability and willingness to undertake and desire for digital transformation projects. Therefore, it is necessary to include the asset-to-liability ratio as a control variable in this study. The calculation method for this indicator is the end-of-period total liabilities divided by the end-of-period total assets.

**Earnings per Share (EPS):** Earnings per share is a crucial indicator for investors and the market to evaluate a company’s value, potentially influencing decisions related to research and development investment, digital transformation, and other relevant matters by management. Controlling for earnings per share can assist in comprehensively considering the overall economic condition of the company. This variable is measured by dividing the net profit attributable to the parent company by the number of ordinary shares issued.

**Shareholding Ratio of the Largest Shareholder (Largest):** This variable assesses the concentration of company ownership by dividing the number of shares held by the largest shareholder by the total number of shares. On one hand, the largest shareholder typically wields significant influence over a company’s decisions, affecting the degree of control over resource allocation, which may drive or hinder the decision-making process of digital transformation. On the other hand, the largest shareholder may influence the company’s risk-bearing capacity. Some shareholders may be more inclined to take on the risks associated with innovation and digital transformation, while others may be more conservative. Therefore, including the maximum shareholder ownership ratio as a control variable in this study helps enhance the internal validity of the research.

**Cash Flow Ratio (Cashflow):** The cash flow ratio reflects the stability of a company’s cash flow. Companies with a high cash flow ratio may possess greater financial flexibility, meaning they have more cash available for research and development and digital transformation, enabling them to more easily cope with changes and challenges brought about by digital transformation. Therefore, it is necessary to include the cash flow ratio as a control variable in this study. This indicator is measured by the ratio of net cash flow from operating activities to total assets.

**Age of the Enterprise (FirmAge):** The age of a company represents its industry-specific experience, which may impact its understanding and adaptability to digital transformation. Younger companies may adapt more quickly to market changes as they may lack deep-seated historical burdens, potentially influencing the speed of implementation of research and development and digital transformation. By controlling for company age, this study can better understand the independent impact of financial subsidies on the speed of digital transformation. This variable is calculated as the natural logarithm of 1 plus the age of the company, expressed as ln(2022—year of establishment + 1).

**Financial Leverage (FL):** The financial leverage indicator reflects the stability of a company in terms of its financial structure. High financial leverage may imply that a company faces a greater debt burden, requiring more payment of interest and financial costs, which could potentially affect whether a company has sufficient funds for digital transformation. Simultaneously, the level of financial leverage may impact a company’s financial decisions, including investment and financing decisions. By controlling for financial leverage, this study can better eliminate interference from other potential factors. The metric for financial leverage is calculated by taking the total of net profit, income tax expenses, and financial costs, and dividing this sum by the aggregate of net profit and income tax expenses.

**Revenue Growth Rate (Growth):** The revenue growth rate reflects the level of market competition. In highly competitive markets, companies may need digital transformation to maintain competitiveness. In this study, including the revenue growth rate as a control variable helps account for the potential impact of business dynamics in digital transformation research. The calculation for this indicator is (current period operating income—previous period operating income) divided by previous period operating income.

### 3.3 Data sources

This study concentrates on the period from 2013 to 2022, focusing on manufacturing companies listed on the Shanghai and Shenzhen A-share markets. The main data sources encompass the Wind database, CSMAR, China Industrial Enterprise Database, and China Economic Network Statistical Database. Several measures were implemented to ensure data reliability: (1) Excluding ST,*S, and PT enterprises throughout the sample period; (2) Eliminating observations from the IPO year. The final dataset comprises 19,395 unbalanced panel data of enterprise-annual observations. Additionally, to mitigate the impact of extreme values, all continuous variables were winsored within the 1st and 99th percentiles. Descriptive statistics of the main variables are provided in Table 1.

**Table 1:** Decriptive statistics.

Variable Type	Variable Name	Symbol	Mean	SD	Min	Max
Explained Variable	digital transformation	*Dig*	1.042	0.966	0	5.615
Explanatory Variable	Financial subsidy	*Sub*	0.163	0.0198	0	0.224
Mediator Variable	R&D investment	*RD*	0.0478	0.0408	0	0.242
Control Variables	Asset-liability ratio	*Lev*	0.396	0.193	0.0083	1.957
	Earnings per share	*Eps*	0.448	1.281	-10.71	49.93
	Proportion of the largest shareholder	*Top1*	32.81	14.09	1.844	89.99
	Revenue growth rate	*Growth*	0.275	3.978	-0.952	429.0
	Years of establishment	*FirmAge*	2.941	0.303	1.609	4.143
	Cashflow ratio	*Cashflow*	0.0510	0.0715	-0.742	0.839
	Financial leverage	*FL*	1.357	19.15	-582.6	2,403

### 3.4 Analysis of correlation

To ensure the reliability and effectiveness of the regression model, it is necessary to conduct a correlation analysis on the main variables of all samples before analyzing the model. The specific results are shown in Table 2. According to Table 2, we can observe a positive correlation between financial subsidies and the digital transformation of enterprises. For the other control variables, the correlation coefficients are all less than 0.5. Furthermore, to address the issue of multicollinearity, we conducted a multicollinearity test, and the results are presented in Table 3.

**Table 2:** Matrix of correlations.

Variables	(1)	(2)	(3)	(4)	(5)	(6)	(7)	(8)	(9)
***(1) Dig***	1.000								
***(2) Sub***	0.085	1.000							
***(3) Lev***	-0.018	0.207	1.000						
***(4) Eps***	0.012	0.072	-0.144	1.000					
***(5) Top1***	-0.061	0.055	-0.022	0.077	1.000				
***(6) Growth***	0.002	0.018	0.026	0.043	0.006	1.000			
***(7) FirmAge***	0.024	0.050	0.112	-0.004	-0.069	-0.002	1.000		
***(8) Cashflow***	-0.064	0.116	-0.179	0.334	0.107	0.001	0.032	1.000	
***(9) FL***	-0.006	0.002	0.007	-0.004	-0.005	0.002	-0.021	-0.015	1.000

**Table 3:** Variance inflation factor.

	VIF	1/VIF
***Cashflow***	1.18	.847
***Eps***	1.144	.875
***Lev***	1.119	.894
***Sub***	1.078	.927
***FirmAge***	1.022	.978
***Top1***	1.021	.979
***Growth***	1.003	.997
***FL***	1.001	.999
**Mean VIF**	1.071	.

Table 3 presents the results of the multicollinearity test for the main variables. It can be observed that the variance inflation factors (VIF) for each variable are all less than 10. This preliminary indication suggests that there is no severe multicollinearity issue among the main variables, which is an important prerequisite for conducting regression analysis.

## 4. Analysis of empirical results

### 4.1 Benchmark result analysis

This study employed a two-way fixed effects model, fixing the dimensions of both sample years and individual entities. Simultaneously, to mitigate the potential impact of outliers and heteroscedasticity on estimation results, we conducted a robust regression analysis on the entire sample. The estimation results using STATA 17.0 statistical software are presented in Table 4.

**Table 4:** Benchmark regression results.

VARIABLES	(1) Dig	(2) Dig
***Sub***	2.696***	1.070***
	(8.02)	(2.94)
**Constant**	0.165***	0.869**
	(2.83)	(1.97)
**Controls**	NO	YES
**Observations**	19,395	19,395
**R-squared**	0.096	0.257
**Number of id**	3,186	3,186
**Firm FE**	YES	YES
**Year FE**	YES	YES

Robust t-statistics in parentheses

*** p<0.01

** p<0.05

* p<0.1

In the first column (1), we did not include control variables in the regression. The results show that financial subsidies significantly contribute to the promotion of enterprise digital transformation. Examining the regression results in the second column (2), the coefficient for financial subsidies (α1) is 1.070, with a significance level of 1%. This indicates that even after incorporating a series of control variables, financial subsidies continue to have a significant positive impact on digital transformation in the manufacturing industry, thereby validating the hypothesis *H*1 in this study.

### 4.2 Robustness test

To enhance the robustness assessment of the previous research results, we conducted a supplementary test of Formula (1), and the corresponding regression results are presented in Table 5.

**Table 5:** Regression results of robustness test.

	(1)	(2)	(3)
VARIABLES	Replace variable	Change sample range	Lag
***Sublevel***	3.951**		
	(2.38)		
***Sub***		3.047***	
		(4.73)	
***L_Sub***			1.415**
			(1.97)
**Constant**	1.004**	0.646	1.448**
	(2.27)	(1.46)	(2.56)
**Controls**	YES	YES	YES
**Observations**	19,395	19,266	15,842
**R-squared**	0.257	0.259	0.243
**Number of id**	3,186	3,181	2,755
**Firm FE**	YES	YES	YES
**Year FE**	YES	YES	YES

Robust t-statistics in parentheses

*** p<0.01

** p<0.05

* p<0.1

#### 4.2.1 Replacement of core explanatory variable

To address the difficulty in horizontally comparing the absolute value of financial subsidies among different enterprises, we introduced the degree of financial subsidies (*Sublevel*) as an alternative core explanatory variable. Whereas, the variable *Sublevel* is measured using the ratio of financial subsidies to total assets of the enterprise. The computation of Sublevel, wherein $Sublevel=\frac{total\;financial\;subsidies}{total\;assets}$. The first column of Table 5 shows that the coefficients of the core explanatory variables remain positive and significant, reaffirming the robustness of the benchmark regression results.

#### 4.2.2 Change the sample range

Due to the presence of extreme values in certain companies that lacked financial subsidies in a given year, it may influence the baseline regression results. To address this issue, we selectively constrained the regression sample, including only those companies with financial subsidy income greater than 0 in the respective year. We conducted a regression based on the filtered data, and the results are shown in the second column of Table 5. It can be observed that the coefficient of the core explanatory variable remains positive and significant, further confirming the robustness of the research results.

#### 4.2.3 Core explanatory variable lagged

On one hand, considering that this study simultaneously controls for year fixed effects and individual fixed effects, it can mitigate endogeneity bias issues caused by omitted variables to a certain extent. However, a more significant endogeneity issue may arise from the bidirectional causality between the explanatory variable and the dependent variable. Put differently, while fiscal support might influence the digital evolution of businesses, the reverse is also plausible: the extent of a company’s digital transformation could in turn impact its chances of securing financial subsidies. On the other hand, the impact of financial subsidies on a company’s digital transformation could exhibit a delay. Therefore, it is necessary to include the lagged one period of financial subsidies (L_Sub) as the core explanatory variable to revalidate the impact of financial subsidies on the digital transformation of enterprises. As shown in the third column of Table 5, the significance and sign of the core explanatory variable have remained essentially unchanged.

#### 4.2.4 Instrumental variable method

Firstly, the Durbin-Wu-Hausman test rejected the hypothesis that the explanatory variable is unrelated to the disturbance factors, indicating some endogeneity in the core explanatory variable of the model. To address potential endogeneity issues such as omitted variables and simultaneity in the baseline regression model, we applied the Two-Stage Least Squares method using the Instrumental Variable (IV) approach to test the robustness of the benchmark regression results. We selected the "mean of financial subsidies excluding the company’s own subsidies in the same province and industry" as the instrumental variable for the core explanatory variable. To reduce data skewness and bring it closer to a normal distribution, we applied a logarithmic transformation to this instrumental variable, calculated as: IV_Sub = ln(1+mean of financial subsidies excluding the company’s own subsidies in the same province and industry). The rationale for choosing this instrumental variable lies in the fact that, on one hand, local government subsidies to enterprises are discretionary, and there is a positive correlation between financial subsidies received by other companies in the same industry within the province and the financial subsidies received by the target enterprise. On the other hand, the financial subsidies received by other companies in the same industry within the same province are unrelated to the digital transformation activities of the target enterprise. The results of the instrumental variable regression are presented in Table 6.

**Table 6:** Regression results of instrumental variable method.

	(1)	(2)
VARIABLES	first stage	second stage
	Sub	Dig
***IV_Sub***	0.0012***	
	(0.000)	
***Sub***		127.0195***
		(10.977)
**Constant**	0.1225***	-17.0387***
	(0.002)	(1.563)
**Controls**	YES	YES
**Observations**	17,580	17,580
**R-squared**	0.168	

Standard errors in parentheses

*** p<0.01, ** p<0.05, * p<0.1

Secondly, as indicated in the second column of Table 6, the coefficient estimates in the second-stage regression are significantly positive at the 1% level. This implies that, after eliminating endogeneity interference, the coefficients of the core explanatory variable remain positive and significant. The impact of local government subsidies on the digital transformation of enterprises remains highly significant. The p-value of the Anderson LM statistic is 0, passing the under-identification test for instrumental variables. Additionally, the Wald statistic is 161.729, exceeding the 10% significance threshold, indicating that the instrumental variable has passed the weak instrument test, reaffirming the validity and rationality of the instrumental variable selection. In summary, the analysis results align with those of the standard regression model, reaffirming the robustness of the results.

### 4.3 Heterogeneity analysis

#### 4.3.1 Enterprise scale

To examine the impact of scale on enterprise digital transformation, this study, based on the classification method in the National Bureau of Statistics’ "Classification Method of Large, Medium, Small and Micro Enterprises (2017)," divided the entire sample into two sub-samples: large enterprises and small and medium-sized enterprises (SMEs). According to the criteria, enterprises with both a workforce of over 1,000 and main business revenue exceeding four hundred million yuan were defined as large enterprises, while the rest were categorized as SMEs. Regression analyses were conducted separately for each sub-sample, and the results are presented in Table 7.

**Table 7:** Regression results of firm size heterogeneity test.

	(1)	(2)
VARIABLES	Big	Small
***Sub***	1.280***	-0.083
	(3.10)	(-0.11)
**Constant**	1.433***	-1.274
	(2.89)	(-0.98)
**Controls**	YES	YES
**Observations**	18,397	998
**R-squared**	0.258	0.205
**Number of id**	3,081	384
**Firm FE**	YES	YES
**Year FE**	YES	YES

Robust t-statistics in parentheses

*** p<0.01, ** p<0.05, * p<0.1

From Table 7, it is evident that financial subsidies significantly promote digital transformation in large enterprises, with a significance level of 1%. However, for SMEs, the coefficient of the impact of financial subsidies on digital transformation even appears negative, though not statistically significant. This may be attributed to the following reasons: On one hand, large enterprises typically have more abundant resources, including financial, technological, and human resources, making it easier for them to invest in digital transformation. Financial subsidies may be more effectively utilized in these large enterprises. In contrast, SMEs, due to their smaller scale, may face greater financial pressure, resulting in the impact of financial subsidies being less significant for them.

On the other hand, large enterprises usually have a higher starting point in terms of technological infrastructure and digitalization levels, making them more likely to benefit from the investments brought about by financial subsidies. Conversely, SMEs may need more effort to establish infrastructure and face greater difficulties in the early stages of digital transformation, making the impact of financial subsidies relatively limited for them. In summary, large enterprises possess superior digital transformation capabilities, leading to a smoother implementation of financial subsidy policies.

#### 4.3.2 Industry type

To examine the impact of industry types on the digital transformation of enterprises, this study, based on the China Securities Regulatory Commission’s 2012 industry classification standard, divides the entire sample into three sub-samples: technology-intensive, asset-intensive, and labor-intensive. Furthermore, drawing on the industry classification method used by Yin in their study on innovation investment and company performance, this study utilizes two indicators—fixed asset ratio (net fixed assets / total assets) and research and development (R&D) expenditure to wage ratio (R&D expenditure / total employee compensation)—as classification criteria [47].

Firstly, the fixed asset ratios and R&D expenditure to wage ratios are calculated for each of the 29 detailed industries. Subsequently, the sample involving these 29 detailed industries undergoes cluster analysis using the Ward’s linkage method. The advantage of this method lies in minimizing the differences within the classified groups while maximizing the differences between groups. The classification that minimizes the sum of squared deviations within each group and is optimal when the number of classes is fixed is chosen.

Furthermore, based on the magnitude of the fixed asset ratios, the sample is classified. Categories with a relatively large fixed asset ratio are defined as capital-intensive industries, indicating a higher importance of capital in these industries. Secondly, based on the magnitude of the R&D expenditure to wage ratio, categories with a higher proportion are classified as technology-intensive industries. This reflects a higher importance of technological research and development relative to labor in these industries, while the remaining categories are classified as labor-intensive industries. The classification results for the 29 detailed industries involved in the sample are presented in Table 8.

**Table 8:** Industry classification results.

Industry	Capital-intensive	Technology-intensive	Labor-intensive
**Subsector code**	C22 C23 C24 C25 C26 C28 C29 C30 C31 C32 C33	C27 C34 C35 C36 C37 C38 C39 C40 C41 C42	C13 C14 C15 C17 C18 C19 C20 C21
**Sample**	5166	11562	2129

The results of the industry-specific heterogeneity regression are presented in Table 9. From Table 9, it can be observed that the effect of financial subsidies on the digital transformation of enterprises varies across industries with different factor proportions. For capital-intensive industries, financial subsidies have a significant positive impact on the digital transformation of non-capital-intensive enterprises, while their impact on capital-intensive industries is not significant. This could be due to the fact that non-capital-intensive enterprises are more likely to benefit from financial subsidies, which may provide additional resources and support, aiding in driving digital transformation. However, capital-intensive industries may prioritize other investments, such as technological research and development or large-scale equipment upgrades, where the role of financial subsidies might be relatively limited.

**Table 9:** Regression results of industry heterogeneity test.

	(1)	(2)	(3)	(4)	(5)	(6)
VARIABLES	Capital-intensive	Non Capital-intensive	Technology-intensive	Non Technology-intensive	Labor-intensive	Non Labor-intensive
***Sub***	-0.124	1.361***	1.343**	0.470	1.043	0.972**
	(-0.30)	(3.03)	(2.35)	(1.13)	(1.52)	(2.29)
**Constant**	2.084***	0.619	0.332	1.886***	1.542*	0.676
	(3.52)	(1.20)	(0.55)	(3.53)	(1.72)	(1.34)
**Controls**	YES	YES	YES	YES	YES	YES
**Observations**	3,717	15,678	11,602	7,793	4,039	15,356
**R-squared**	0.230	0.270	0.290	0.223	0.231	0.268
**Number of id**	607	2,630	1,999	1,257	655	2,580
**Firm FE**	YES	YES	YES	YES	YES	YES
**Year FE**	YES	YES	YES	YES	YES	YES

Robust t-statistics in parentheses

*** p<0.01

** p<0.05

* p<0.1

For technology-intensive industries, financial subsidies are positively correlated with the digital transformation of technology-intensive enterprises at a 1% significance level. This aligns with our subjective judgment, as technology-intensive enterprises rely more on advanced technology and research and development, and financial subsidies can provide funds to expedite the process of digital transformation. However, in non-technology-intensive enterprises, the positive impact of financial subsidies is not significant. This could be because these enterprises benefit less from digital transformation or other factors such as market competition and management strategies have a more significant impact on their digital transformation.

For labor-intensive industries, financial subsidies significantly promote the digital transformation of non-labor-intensive enterprises, with a coefficient of 0.972 and significance at the 5% level. However, the impact of financial subsidies on the digital transformation of labor-intensive enterprises is not significant. Non-labor-intensive enterprises may rely more on investments in high-tech equipment and advanced technology, and financial subsidies provide additional financial support, making it easier for these enterprises to acquire and apply digital technology. Additionally, non-labor-intensive enterprises typically prioritize technological innovation and high value-added industry chains, and digital transformation is a critical means to achieve these goals. Financial subsidies can serve as part of the innovation drive, stimulating enterprises to innovate digitally through financial support.

However, labor-intensive enterprises may focus more on aspects such as human resources and production efficiency in their operations. Meanwhile, some labor-intensive industries may face greater challenges in digital transformation, as their operations may be more dependent on traditional manual operations, and digital transformation may involve larger changes and adaptation periods. It is worth noting that the design of financial subsidy policies often fails to fully consider the unique needs and challenges of labor-intensive enterprises, resulting in a relatively weaker impact on their digital transformation. Through this analysis, it is evident that different types of enterprises exhibit variations in their paths to digital transformation, and financial subsidy policies may need to be more specifically tailored to accommodate the diverse needs of different industries.

### 4.4 The degree of R&D investment as the mediating role of the incentive effect

The results of the mediation effect model are presented in Table 10. In the first column, the regression based on Formula (1) confirms that financial subsidies directly promote the digital transformation of manufacturing enterprises, as mentioned earlier. Moving to the second column, the explanatory variable now becomes the enterprise’s R&D investment (*RD*). The coefficient of the financial subsidy (*Sub*) variable (β_1_) is 0.071, with significance at the 1% level, indicating a significant impact of financial subsidies on the enterprise’s R&D investment. This result supports Hypothesis *H*_2_*a*. In the third column, after controlling for the influence of R&D investment, the coefficient of the subsidy variable (*Sub*) is 1.005, with significance at the 1% level. Additionally, after controlling for the impact of financial subsidies, the coefficient of R&D investment (*RD*) is 0.961, significant at the 5% level. These findings demonstrate that R&D investment plays a significant role in stimulating the digital transformation of enterprises, supporting Hypothesis H2b. Since both β_1_ and *c*_2_ are significantly positive, and *c*_1_(1.005)<α_1_(1.070), this suggests that investment in research and development acts as a partial intermediary in the relationship between financial subsidies and the stimulatory impact of digital transformation on manufacturing firms. The results in Table 10 provide confirmation for Hypothesis *H*_2_.

**Table 10:** Mediating effect test regression results.

	Formula (1)	Formula (2)	Formula (3)
VARIABLES	Dig	RD	Dig
***RD***			0.916**
			(2.46)
***Sub***	1.070***	0.071***	1.005***
	(2.94)	(4.05)	(2.78)
**Constant**	0.646	0.051***	0.602
	(1.46)	(2.77)	(1.37)
**Controls**	YES	YES	YES
**Observations**	19,266	19,266	19,266
**R-squared**	0.259	0.076	0.260
**Number of id**	3,181	3,181	3,181
**Firm FE**	YES	YES	YES
**Year FE**	YES	YES	YES

Robust t-statistics in parentheses

*** p<0.01

** p<0.05

* p<0.1

## 5. Further analysis

### 5.1 Theoretical analysis of the moderating effect of marketization level

The degree of marketization exerts an influence on both factor markets and product markets, thereby yielding various economic effects on corporate digital transformation. These effects encompass scale, competitive, demonstration, and spillover effects, collectively modulating the incentivizing impact of financial subsidies on digital transformation initiatives.

To begin, regions characterized by a high degree of marketization are conducive to nurturing the requisite environmental and legal conditions for digital transformation [48]. This environment facilitates manufacturing enterprises receiving subsidies to more easily procure essential factors from the market, thus enhancing resource and factor allocation efficiency [49], thereby stimulating digital transformation. Furthermore, regions with high marketization exhibit reduced market barriers, providing businesses with expanded market opportunities, enabling them to innovate in digital technology based on market demand [50]. Additionally, highly marketized regions possess well-developed product markets with intense competition, increasing market information transparency [51], creating a favorable environment for digital transformation. In highly marketized regions, product prices are market-determined, compelling subsidized enterprises to undertake digital transformation in pursuit of profit maximization rather than diverting subsidy funds for other purposes. Simultaneously, the developmental atmosphere and competitive pressure in highly marketized regions compel enterprises to upgrade and adapt to the ever-evolving digital environment and consumer behavior [52]. Moreover, regions with advanced marketization processes boast more successful digital technology application cases, with strategic decision-makers exhibiting a heightened level of digital awareness and astute judgment regarding the prospects of digital technology applications. This is conducive to deepening the application of digital technology by enterprises. Furthermore, such regions facilitate easier access to highly skilled labor, thereby enhancing the productivity effects of digital technology [53].

The process of marketization has a regulatory effect on the relationship between financial subsidies and research and development (R&D) investment. Liu and Xu observed that in areas where market forces are more dominant, there tends to be fiercer competition among businesses, coupled with a more robust system of property rights protection [54]. In order to pursue competitive advantages and enhance core competitiveness, enterprises in these places will be more active in R&D investment. Tang’s research findings indicate that in regions with a higher level of marketization, governmental actions exert a more substantial positive influence on the research and development investments made by enterprises [55]. However, after empirically examining the data of China’s high-tech enterprises, Liu concluded that government subsidies can promote enterprises’ R&D investment to a greater extent in areas with a higher degree of marketization and relatively limited government subsidies [56]. Numerous studies affirm the regulatory role of marketization processes. This is because in regions with relatively high levels of marketization, the institutional environment is more favorable for the comprehensive development of enterprises. This includes more developed financial markets, stronger property rights protection, and a more robust professional manager market. These factors collectively drive subsidized enterprises to increase their R&D investments [6]. Furthermore, highly marketized regions possess more mature market mechanisms, which play a crucial role in external governance, preventing subsidized enterprises from misallocating funds to activities unrelated to digital transformation, thus ensuring the efficient utilization of financial subsidy funds [8]. On the other hand, a fair and highly competitive environment necessitates more stringent requirements for market participants [57]. Over time, enterprises that solely engage in low-cost imitation and plagiarism will inevitably be eliminated by the market forces. This undoubtedly incentivizes enterprises to pursue technological research and innovation [58], thereby fostering increased investment in research and development [59]. Furthermore, a higher degree of regional marketization corresponds to a more robust financial system, which can alleviate the external financing constraints faced by innovative entities. Within such a market environment, enterprises are presented with greater opportunities to secure commercial loans [60]. Additionally, an elevated level of regional marketization is associated with enhanced quality of enterprise information disclosure. This significantly reduces the cost of information for external investors while improving prospects for enterprises to attract external investments [61].

There is currently no direct empirical evidence linking marketization levels to the effectiveness of R&D investment in driving digital transformation in manufacturing enterprises. However, our analysis suggests possible regulatory pathways. These pathways may influence how marketization processes affect the relationship between R&D investment and digital transformation. On one hand, in highly marketized industries, competition is fiercer, and companies need to invest more in R&D to maintain a competitive advantage, thus promoting digital transformation. On the other hand, marketization processes drive companies to focus more on innovation, which, in turn, encourages greater R&D investment and subsequently promotes digital transformation. Furthermore, marketization processes influence the availability and accessibility of resources for companies, making it easier for them to increase R&D investment, thereby promoting digital transformation. Additionally, marketization processes achieve this effect by influencing the strategic decisions of corporate management, guiding their approach to digital transformation. In highly marketized regions, resource allocation follows market principles, leading corporate management to view digital transformation as part of their long-term strategic planning. In contrast, in regions with lower levels of marketization and limited resources for strategic changes, corporate management may perceive digital transformation as a short-term “showcase project” rather than a long-term strategic arrangement [62].

### 5.2 Empirical testing

Building upon the aforementioned analysis, the moderating variable in this study is the degree of marketization (*Market*). The marketization index outlined in the “China Provincial Marketization Index Report” is utilized to gauge the level of marketization in the region where the enterprises are located. This index is structured around five fundamental aspects. It evaluates the dynamics between governmental policies and market forces, alongside the evolution of privately-owned enterprises. Furthermore, it assesses the maturity of both product and factor markets. Additionally, the index examines the progress in market intermediary frameworks and the effectiveness of the legal system. Its credibility stems from its comprehensive, scientifically-grounded, and trustworthy methodology. Compared with other ways to measure the degree of marketization development, this index is more consistent with the situation of China’s localized market. In this study, the original index has been scaled down by a factor of 100. This data transformation ensures that the variance of variables and estimated standard errors remain unchanged. Simultaneously, it addresses differences in the magnitude of variables, making regression coefficients less sensitive for ease of observation and comparison.

On the basis of Formulas (1), (2), and (3), we introduced the interaction terms (*Sub*_*Market*) between marketization level (*Market*) and financial subsidies (*Sub*), as well as the interaction term (*RD*_*Market*) between marketization level (*Market*) and the level of research and development (RD). Furthermore, we constructed a moderated mediation model to examine the moderating role of marketization level in both the direct incentive effect and the two stages of the indirect incentive effect.

The results of the moderated mediation model are presented in Table 11. The first column represents the baseline regression model for comparison with the moderated mediation model. To address the issue of high collinearity between the interaction terms and explanatory variables or moderating variables, the study centralized the independent and moderating variables by subtracting the sample mean.

**Table 11:** Regression results of moderation effect test.

	(1)	(2)	(3)	(4)
VARIABLES	Dig	Dig	RD	Dig
***Sub***	1.070***	1.322***	0.082***	1.096***
	(2.94)	(3.33)	(3.97)	(2.82)
***Market***		3.178**	-0.029	3.009**
		(2.58)	(-0.55)	(2.47)
***Sub_Market***		45.197**	2.196**	30.372*
		(2.45)	(2.26)	(1.65)
***RD***				0.902**
				(2.32)
***RD_Market***				145.753***
				(7.09)
**Constant**	0.869**	0.572	0.067***	0.761*
	(1.97)	(1.28)	(3.62)	(1.76)
**Controls**	YES	YES	YES	YES
**Observations**	19,395	19,395	19,395	19,395
**R-squared**	0.257	0.258	0.072	0.267
**Number of id**	3,186	3,186	3,186	3,186
**Firm FE**	YES	YES	YES	YES
**Year FE**	YES	YES	YES	YES

Robust t-statistics in parentheses

*** p<0.01

** p<0.05

* p<0.1

Moving to the second column, it examines the moderating effect of marketization level in the direct incentive effect of financial subsidies on enterprise digital transformation. The coefficient for the interaction term (*Sub*_*Market*) between marketization level and financial subsidies is 45.197, and it is significant at the 5% level. This is consistent with the theoretical analysis above, indicating that marketization level positively moderates the direct incentive effect of financial subsidies on enterprise digital transformation. In other words, the higher the marketization level, the stronger the promotion effect of financial subsidies on digital transformation.

Moving to the third column, the analysis focuses on examining how the level of marketization influences the dynamic between digital transformation and investment in research and development. It aims to understand the regulatory impact of marketization within this context. The coefficient for the interaction term (*Sub*_*Market*) between marketization level and financial subsidies is 2.196, and it is significant at the 5% level. This suggests that marketization level positively moderates the relationship between firm’s research and development investment and digital transformation. With the rise in marketization levels, the effects of investing in research and development on facilitating digital transformation grow more distinct.

In the analysis presented in the fourth column, the interaction term (RD_Market) combining research and development investment with marketization level shows a coefficient of 145.753. This value is significantly positive, especially notable at the 1% level. This finding suggests that as marketization level escalates, its positive influence on the bond between corporate investment in research and development and digital transformation becomes more evident. The increasing impact of R&D investment on digital transformation is particularly marked with rising marketization levels. The significant positivity of the interaction coefficients 2.196 (Sub_Market) in the third column and 145.753 (RD_Market) in the fourth column implies that the marketization level exerts a constructive moderating effect throughout both stages of the mediation process.

## 6. Research conclusions and policy recommendations

### 6.1 Conclusion

This study employs panel data collected from manufacturing companies listed in China’s Shanghai and Shenzhen A-share markets over the period from 2013 to 2022. It empirically examines the influence of financial subsidies on the digital transformation of manufacturing companies. Furthermore, it explores the mediating role of R&D investment and the moderating impact of the degree of marketization, utilizing an adjusted mediation effect model. The key findings of the study can be summarized as follows:

1. Financial subsidies, in general, significantly propel the digital transformation of manufacturing enterprises.
2. The impact of financial subsidies on the digital transformation varies significantly based on enterprise types. Notably, financial subsidies have a significant stimulating effect on large enterprises, non-asset-intensive enterprises, technology-intensive enterprises, and non-labor-intensive enterprises. However, the stimulating effect on small and medium-sized enterprises, asset-intensive enterprises, non-technology-intensive enterprises, and labor-intensive enterprises is not significant.
3. R&D investment plays a crucial role as an intermediary in the digital transformation process. An increase in financial subsidies results in a substantial rise in R&D investment, subsequently motivating enterprises to engage in digital transformation.
4. The degree of marketization acts as a regulator between financial subsidies and the digital transformation of manufacturing enterprises. An enhanced degree of marketization in the enterprise’s region positively adjusts both the direct and indirect incentive effects of financial subsidies on digital transformation.

The study systematically expounds the influence of marketization level, mechanisms, and regulation. This research contributes in several ways: First, this research enriches the existing measures of digital transformation. It combines manual curation and computer text mining methods to select digital transformation indicators for assessing the level of digital transformation in manufacturing enterprises. Second, it enhances the research on the mechanisms of financial subsidies’ impact on digital transformation. Third, this research provides empirical evidence on how market participation affects the effectiveness of financial subsidies. Finally, this research assists policymakers in comprehensively understanding the impact of financial subsidy policies on different types of enterprises.

### 6.2 Policy implications

In light of the research findings, several recommendations are proposed:

Enhancing Financial Subsidy Policy Sustainability. As discussed in this paper, enhancing the sustainability of financial subsidy policies is crucial for promoting the digital transformation of manufacturing enterprises. Therefore, it is advisable to cautiously increase financial subsidies for manufacturing enterprises to alleviate the burden of digital transformation and foster further development in this area. However, considering the current economic downturn and substantial tax reductions, ensuring financial sustainability is also an urgent consideration. The government can establish a more flexible and transparent financial support mechanism to ensure the efficient use of subsidy funds. This includes clear fund usage regulations, regular fund audits, and the establishment of an effective regulatory system to prevent the misuse or waste of subsidy funds. Increased transparency and compliance will help build trust in financial subsidy policies within society, ensuring that funds are used effectively for the intended purpose of digital transformation. Furthermore, to effectively implement enterprise subsidy policies, increasing the visibility of financial and taxation policies and providing comprehensive guidance on relevant policies are essential.

Enhancing the relevance of financial subsidy policies. This study shows that although financial subsidies play a crucial role in the market, they do not significantly promote the digital transformation of small and medium-sized enterprises, asset-intensive enterprises, non-technology-intensive enterprises and labor-intensive enterprises. In view of this problem, it is recommended that the government strengthen communication and cooperation with all kinds of enterprises to better understand their pain points and needs in digital transformation. Through regular symposia and enterprise research, the government can have a more comprehensive understanding of the practical problems faced by enterprises in the process of digital transformation, and adjust the financial subsidy policies in a targeted manner. Such a participation mechanism can better guarantee the relevance and pertinence of policies. In addition, the government can also set up a special consulting agency or digital transformation guidance organization to provide customized advice and support to enterprises to help them better develop digital strategies. All in all, it is necessary to consider the types and needs of enterprises comprehensively. Through customized policies, in-depth communication and cooperation, strengthen the participation of industrial organizations and other ways to build a systematic and comprehensive support system. This will play a crucial role in promoting the digital transformation of enterprises.

Enhancing the Level of Regional Marketization. The research presented in this paper illustrates that the degree of marketization in the region where manufacturing enterprises operate positively impacts the effectiveness of financial subsidies in driving enterprise digital transformation. In particular, a higher degree of marketization enhances the policy effect of financial subsidies. Therefore, it is crucial to maintain the market’s central role in propelling the transformation and development of manufacturing enterprises. The allocation of financial subsidies should avoid unnecessary interference with the market, and preferential policies should align with market demands. Simultaneously, it is essential to improve the market-based allocation mechanism for traditional production factors such as labor, capital, and technology, as well as for emerging production factors like data. Facilitating equitable access to a variety of production factors for market participants will enable them to effectively undertake digital transformation initiatives. Notably, data is a novel production element, and its market-oriented allocation presents challenges. To address this, it is recommended to initiate pilot projects and coordinate comprehensive reforms to effectively manage the market-based allocation of data.

## Supporting information

S1 Data (DTA)
