# Correction: Novel enterprises digital transformation influence empirical study

**DOI:** 10.1371/journal.pone.0328759

**Published:** 2025-07-21

**Authors:** Xiaowen Sun, Wenjing Sun, Zheng Wang

There are errors in the article title. The correct title is: Fiscal Subsidies and Digital Transformation: Empirical Evidence from Manufacturing Enterprises. The correct citation is: Sun X, Sun W, Wang Z (2024) Fiscal Subsidies and Digital Transformation: Empirical Evidence from Manufacturing Enterprises. PLoS ONE 19(1): e0296693. https://doi.org/10.1371/journal.pone.0296693.
